# Soft hybrid intrinsically motile robot for wireless small bowel enteroscopy

**DOI:** 10.1007/s00464-021-09007-7

**Published:** 2022-01-31

**Authors:** Hamza Khan, Afshin Alijani, Craig Mowat, Alfred Cuschieri

**Affiliations:** 1 School of Medicine, University of Dundee, Dundee, DD21FD UK; 2 Ninewells Hospital, Dundee, UK

**Keywords:** Enteroscopy, Wireless capsule endoscopy (WCE), Active vibration-induced locomotion, Active transit in small bowel

## Abstract

**Background:**

Difficulties in establishing diagnosis of small bowel (SB) disorders, prevented their effective treatment. This problem was largely resolved by wireless capsule endoscopy (WCE), which has since become the first line investigation for suspected SB disorders. Several types of WCE pills are now used in clinical practice, despite their limitations and complications. WCE pills are large, rigid and immotile capsules. When swallowed, they provide SB enteroscopy downloaded to a data logger carried by the patient. Most of the complications of WCEs result from lack of intrinsic locomotion: incomplete examination, capsule retention and impaction within strictures. In addition, the rigid nature and size of current generation of WCE pills is accompanied by 0.1% inability to swallow the pill by patients with normal esophageal motility.

**Methods:**

The aim of this communication is to describe the initial prototype, P_1_, which is thinner and slightly longer than the current generation of WCEs. In addition, it exhibits intrinsic active locomotion, produced by vibrating silicon legs. These generate a controlled-skid locomotion on the small bowel mucosal surface, rendered slippery by surface mucus and intraluminal surfactant bile salts. We demonstrate the mechanism responsible for the active locomotion of P_1_, which we consider translatable into a working prototype, suitable for further R&D for eventual clinical translation.

**Results:**

The shape and attachment of the rubber vibrating legs to vibrating actuators, have been designed specifically to produce a tight clockwise circular motion. When inserted inside a circular tube in vitro of equivalent diameter to human small intestine, the intrinsic circular clockwise motion of P_1_ translates into a linear locomotion by the constraints imposed by the surrounding circular walls of SB and rest of the gastrointestinal tract. This design ensures device stability during transit, essential for imaging and targeting lesions encountered during the enteroscopy. We preformed two experiments: (i) transit of P_1_ through a phantom consisting of a segment of PVC tube placed on a horizontal surface and (ii) transit through a transparent slippery nylon sleeve insufflated with air. In the PVC tube, its transit rate averages 15.6 mm/s, which is too fast for endoscopy: whereas inside the very slippery nylon sleeve insufflated with air, the average transit rate of P_1_ is reduced to 5.9 mm/s, i.e., ideal for inspection endoscopy.

**Conclusions:**

These in-vitro experiments indicate that the P_1_ hybrid soft robot prototype has the potential specifically for clinical translation for SB enteroscopy.

**Supplementary Information:**

The online version contains supplementary material available at 10.1007/s00464-021-09007-7.

The introduction of wireless capsule endoscopy (WCE) [1–6] in 2000 enabled early detection of common SB disorders, e.g., occult bleeding and tumors. However, despite considerable progress in the technology for WCE, there remains several unresolved issues, the most important being lack of active controlled locomotion [7]. All the WCE devices are essentially rigid passive pills that rely on gravity, assisted by the peristaltic contractions for transit through the gastrointestinal tract (GIT). The SB consists of a uniform hollow visceral tube disposed in smooth circular loops, situated in the center of peritoneal cavity, and surrounded by the colon.

All the current WCE devices are prone to capsule retention, defined as ‘*the Pill remains in the GIT for more than 2 weeks or requires active removal*’, with a reported incidence of 5% [8]. Furthermore, the capsule fails to reach the caecum, i.e., does not complete the enteroscopy in 20% [9], the reason being due to passive transit of WCE pills. Moreover, the transit of all current WCE devices is both uncontrolled and unpredictable, without any ability to adjust position and orientation [10] including stop-start-reverse guidance, essential for detection and biopsy procurement of suspect lesions. All reported types of WCE locomotion [11–17] have major limitations, either by use of active controlled propulsion requiring large external equipment for magnetic locomotion, or intrinsic device locomotion by means of potentially traumatic appendages, e.g., paddles that add to the bulkhead size. The Dundee Soft Robotics group, initially funded by the Engineering and Physics Science Research Council (EPSRC) of the UK and subsequently the Wellcome Trust Innovation Scheme, embarked on a soft hybrid robotic device to address the issues and complications of WCE pills.

This paper reports on the first Prototype P_1_ of small bowel soft robotic enteroscope (SOFTIE) and its intrinsic locomotion. The physiological reason for this type of skid locomotion relates to intraluminal mucosal surface of the SB, rendered slippery by a surface layer of mucus and surfactant bile salts.

The SOFTIE concept is illustrated in Fig. 1. As can be seen, initially power is provided to SOFTIE by an active tether, but this is detached when the device enters the duodenum. Thereafter, SOFTIE relies on its on-board batteries for its power supply to complete the enteroscopy followed by descending colonoscopy. We have adopted a reverse engineering approach directed towards imparting flexibility and increased ingestibility to overcome issues encountered clinically by all current WCE pills: swallowing difficulties and lack of active locomotion. These have been replaced by a soft segmented intrinsically motile, narrower (< half diameter) but slightly longer than the current generation of WCE pills.

**Fig. 1:**
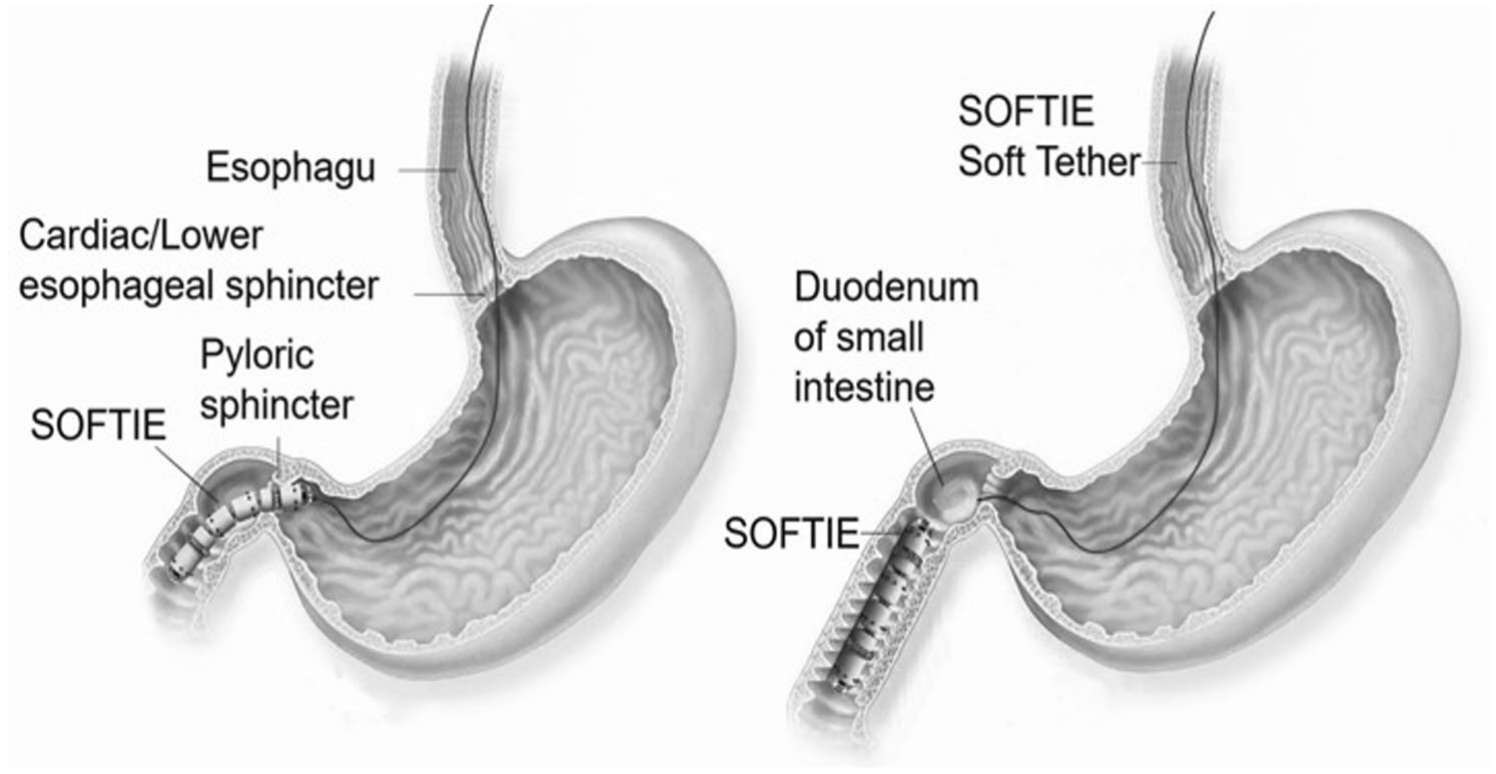
SOFTIE robot (left) before and (right) after detachment of power tether on entry into duodenum

In the next section, we describe the vibrating mechanism underpinning the locomotion of the prototype P_1_ and demonstrate how the rubber legs attached to vibrating motors produce on activation, a *tight clockwise circular motion*. When placed inside circular polyvinyl chloride (PVC) tube or air insufflated slippery nylon sleeve, the *tight circular clockwise locomotion* is constrained by the circular walls of the PVC tube/air insufflated slippery nylon sleeve into linear locomotion. The added benefit of this configuration is provision of device stability, enabling smooth transit until SOTIE exits the PVC tube or air insufflated slippery sleeve, when its intrinsic clockwise locomotion is restored. The clinical benefits of such locomotion are twofold: (i) device stabilization enabling improved targeting and imaging of lesions encountered during the enteroscopy, (ii) improved precision of device and lesion location essential for biopsy procurement.

## Materials and methods

The progress achieved since adopting the reverse engineering R & D approach has been such as to confirm the significant potential for eventual clinical translation of P_1_ prototype. In essence, the reversed engineering approached, entailed splitting the WCE pill into an anterior and posterior sections; followed by splitting each section longitudinally to enable insertion of functional components. Thereafter, the two sections (anterior and posterior) are joined by a flexible intermediate tubular segment forming a highly flexible, easy to swallow tri-segment hybrid soft robot.

In the next subsection, we describe the nature of vibrating mechanism underpinning the locomotion of P_1_.

### Choice of vibration actuators

**Fig. 2:**
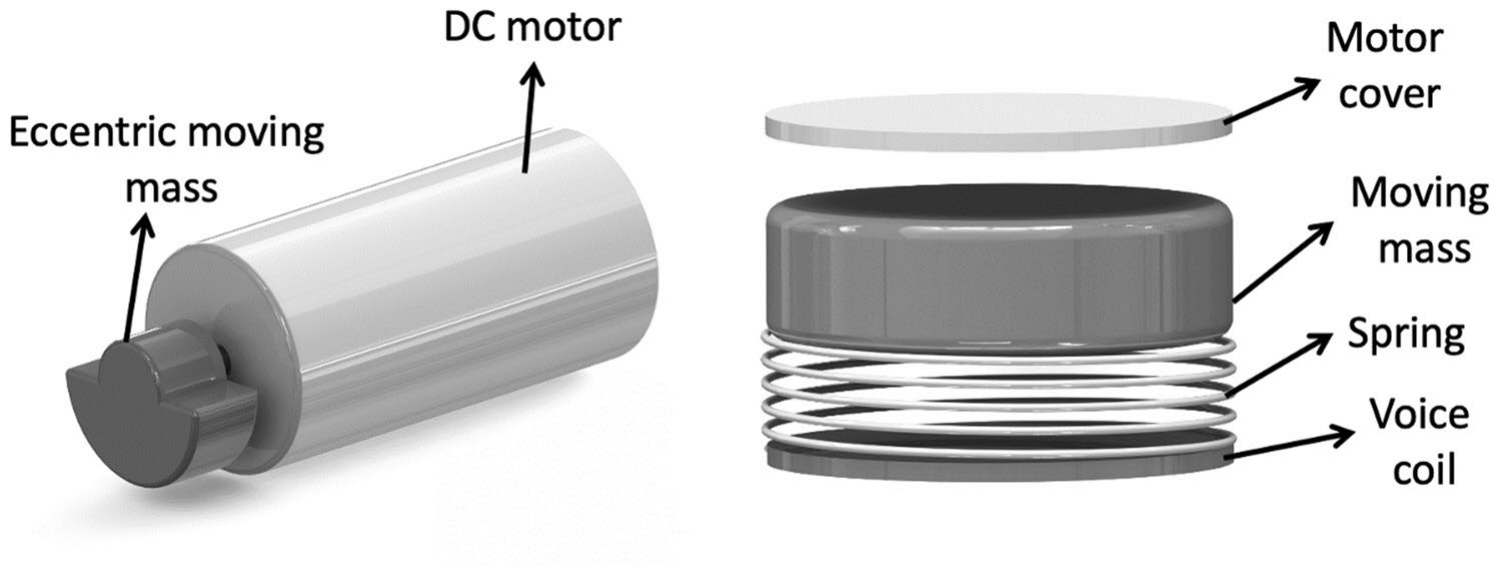
The vibrating mechanism (left): Eccentric rotating mass (ERM), (right): linear resonant actuator (LRA)

The initial modeling of this vibration-induced locomotion involved use of traditional eccentric rotating mass vibration motors (ERMs). These consist of a small eccentric mass on a DC motor, which as it rotates, creates a force that produces vibrations. Its working principle is shown in Fig. 2 (left).

Subsequently, we explored use of linear resonant actuators (LRAs) because of their faster response time and longer life span compared to ERMs. As illustrated in Fig. 2 (right), these produce vibrations by a moving magnetic mass connected to a spring. When electric current is applied, it induces an electromagnetic field within the voice coil that drives the magnetic mass up and down causing displacement of the LRA and hence, the vibration force. LRAs can be operated within a narrow frequency range to optimize their power consumption. Additionally, an alternating current can be used to produce the magnetic field, thereby further reducing power consumption. Both these vibrating mechanisms are capable of intrinsic forward/backward movement of P_1_ that is dependent on the direction of the vibrating legs. In next subsection, we provide details of the special mounting of the rubber legs to vibrating actuators specifically to *realize tight circular locomotion generated* by the vibrating legs.

### Leg design

**Fig. 3:**
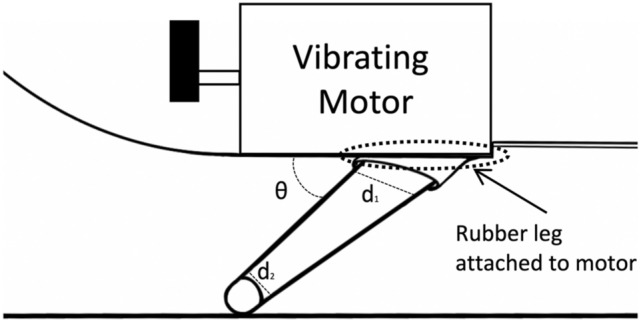
The leg P1 robot is tapered, *θ* defines the mounting angle between motor and leg. Whereas d_1_, d_2_ are variations of thickness along the leg

The rubber silicon legs are the important translating components underpinning the intrinsic active locomotion of P_1_. The mounting angle *θ* and stiffness along the longitudinal axis of the vibrating legs are essential design features that require optimization. Both stiffness & vibration frequency of these legs define its forward speed [18, 19]. In essence, the leg design of P_1_ is based on three parameters: (i) stiffness of the silicon rubber legs, (ii) taper of legs from attachment to motor to the SB mucosal surface and (iii) mounting angle *θ* between the attachment point to vibrating motor and longitudinal axis of silicon leg. As shown in Fig. 3, when legs touch the ground (i.e., mucosal surface of SB), the mounting angle is *θ* and the proximal (d_1_) and distal ends (d_2_) are spatially opposite different thicknesses of the tapered legs.

**Fig. 4:**
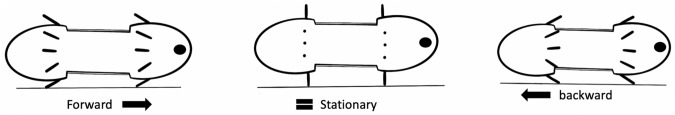
Demonstration P_1_ legs direction for forward, stationary and reverse transit of the P_1_ robot. The black oval shape indicates the front dome of P_1_

After various simulations/ iterations, P_1_ legs were designed to be thicker at the attachment points and tapering down towards the distal end touching the ground (i.e., mucosal surface of SB). For each P_1_ leg, the diameter d_1_ is always greater than d_2_. The mounting angle *θ* defines direction of movement of the P_1_ robot. Thus, for forward movement, the angle must range between 45° and 70°. Similarly, for reverse transit of P_1_, *θ* should range between 135° and 160°. The P_1_ legs vibrate in a static mode when their mounting angle lies between 80° and 100°. Figure 4 (above) illustrates, the precise position of the legs for forward, reverse, and active static locomotion of the robot (active static locomotion is required during procurement of biopsy). Currently leg orientation for forward, reverse, and active static locomotion is only possible by changing manually the position of legs. When the high-level control which will be added to next version P_2_ robot, the leg orientations required for the different mounting angle *θ* will be controlled actively.

## Results

**Fig. 5:**
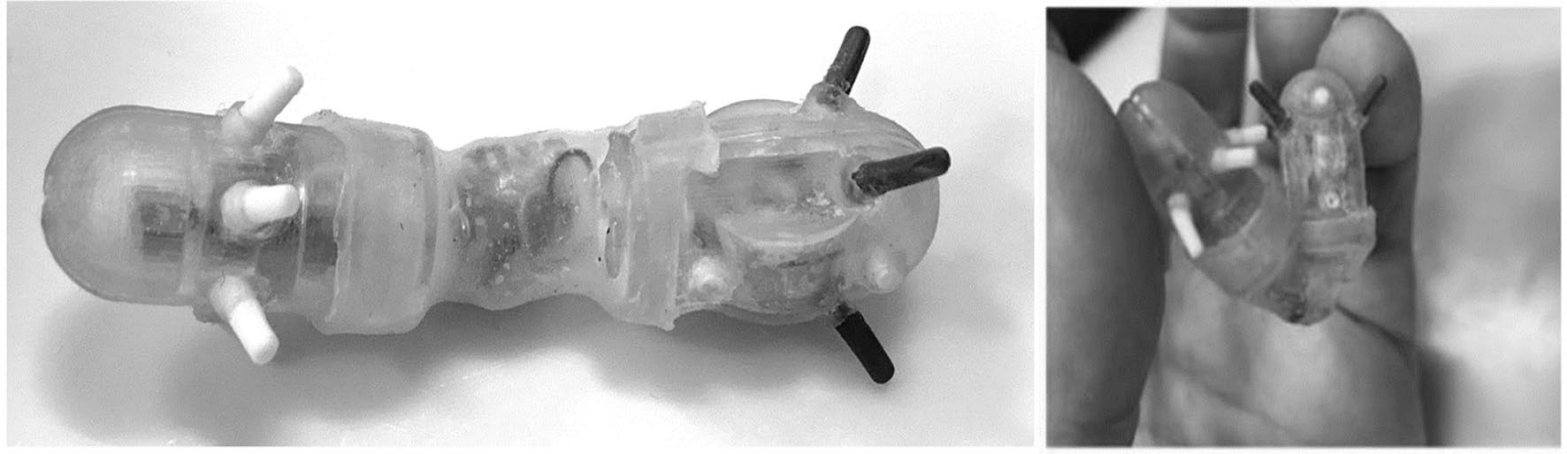
SOFTIE robot P_1_ prototype: (left)  has two components linked by soft Ecoflex bridge, the third component; (right) demonstrates the flexible nature of the device by extreme bending. The white legs belong to anterior segment and the black legs belong to posterior segment (all legs are made from the same silicon)

The current hybrid P_1_ prototype is illustrated in Fig. 5. It consists of three segments; two are rigid, fabricated by 3D printing from plastic ABS (Acrylonitrile Butadiene Styrene) and an intervening flexible silicon connecting segment between the anterior and posterior segments. The wall of each rigid segment is 1 mm thick; its two halves are press fitted to restore their circular shape after insertion of components. The connecting flexible tubular segment is constructed from Ecoflex™ 00–30 and is glued to both rigid segments by Loctite™. The Ecoflex segment contains the electronics, whereas rigid anterior segment the vibration actuator, and posterior segment, the button battery. The total length P_1_ is 30 mm with a diameter of 8 mm. Its vibration actuator (6 mm diameter) has a vibration amplitude of 1.5 G. The silicon used for the legs has a density 110 kg/m^3^, Young’s modulus 1.1 × 10^6^ Pa and a Poisson’s ratio 0.49. In the P_1,_ the mounting angle *θ* of each pair of six legs is 58^◦^. The P_1_ prototype has open loop control.

**Fig. 6:**
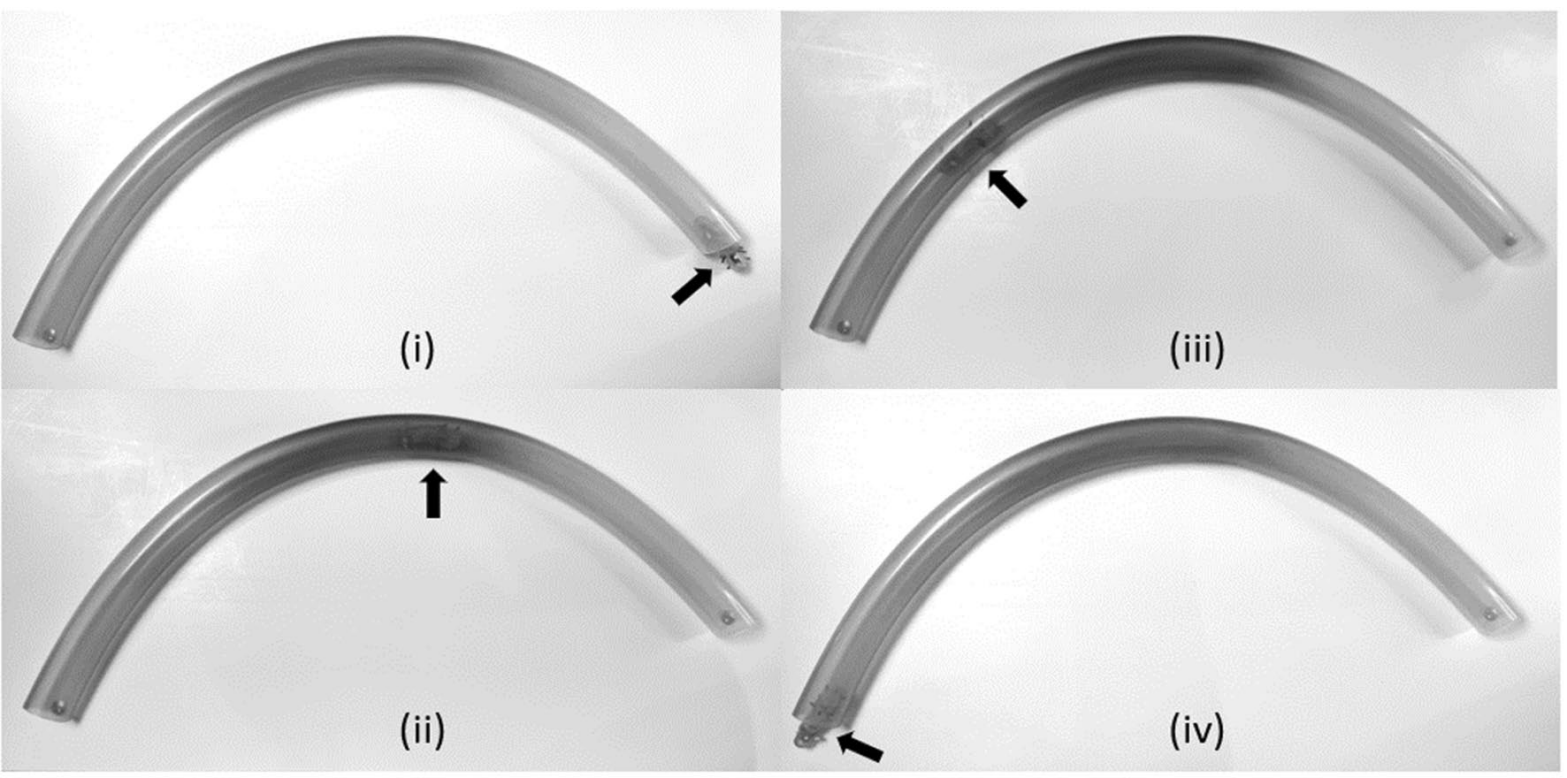
Series of screen shots during tests on SOFTIE prototype P_1_ when tested transiting inside 650 mm long PVC tube

**Fig. 7:**
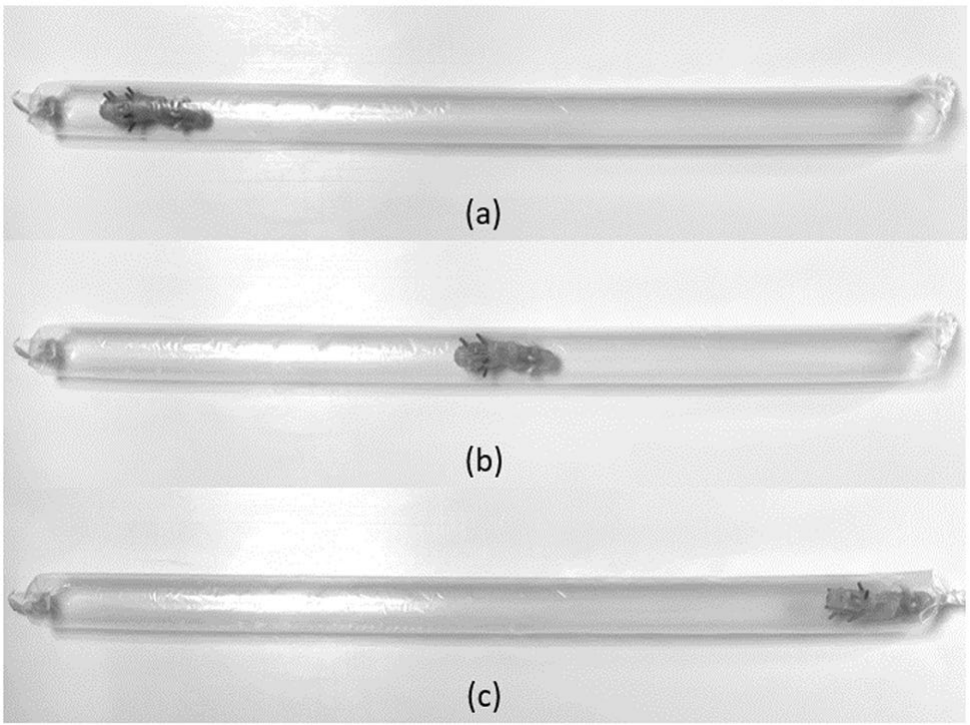
Screen shots during transit tests of SOFTIE prototype P_1_ inside 450 mm long inflated nylon sleeve

We preformed two experiments: (i) transit of P_1_ through a PVC tube placed on horizontal surface and (ii) transit through a slippery transparent nylon sleeve partially insufflated with air. Videos are available for the experiments by Video link in Appendix A. During these experiments, P_1_ transits through 12 mm (inner diameter) by 650 mm length PVC tube shown in Fig. 6. The P_1_ legs are designed such that the locomotion produced is always in a clockwise direction forming a tight circle with a diameter that approximates to length of robot. With these parameters, when the P_1_ is inserted into the PVC tube, it is constrained by the surrounding circular walls, resulting in change of circular to linear locomotion. When it exits from the PVC tube placed on horizontal surface, it resumes its intrinsic circular clockwise motion [Fig. 6 (iv)], thereby it turns right and continues to move along the outer aspect of the PVC tube placed on a horizontal flat surface. The forward speed of P_1_ depends on the vibration frequency and friction of the surface on which it is moving. In PVC tube, its transit rate is therefore fast, averaging 15.6 mm/s. Whereas inside the very slippery air insufflated nylon sleeve (shown on Fig. 7), the transit rate of P_1_ is reduced to 5.9 mm/s, ideal for inspection endoscopy.

The active locomotion in current P_1_ version provides robust experimental confirmation enabling safe prediction that following detachment of the power tether and based on the current transit speed, the P_1_ version reported in this communication, would exhibit sufficient power to enable descending colonoscopy after completion of the SB enteroscopy before exiting through the anus. Furthermore, it is possible from data obtained from these experiments that the future realization of whole gut endoscopy from mouth to anus is feasible.

## Discussion

The primary objective of *P*_*1*_ has been to substitute the rigid immotile WCE devices with a hybrid soft flexible, tri- segment robotic equivalent, thinner and slightly longer that WCE pills, hence easier to swallow by patients and with sufficient transit speed to complete SB enteroscopy and descending colonoscopy before existing through the anal canal. The novel SOFTIE patent application has been filed at the UK Intellectual Property Office. The date of filing of the patent application is 22 July 2021 and the application number is 2110576.2. The tight circular motion is ideal for the entire gastrointestinal tract which is essentially tubular. When the P_1_ reaches the SB, the intrinsically circular motion is constrained by circular surrounding walls, translating into linear locomotion with the added advantage of device stabilization that is essential for both imaging and location of suspect lesions. Although we have achieved all the objectives of P_1_ prototype, we stress that we are at the beginning of R&D process towards realization of a functioning substitute to WCEs. Over the next 3 years, we have an on-going R&D program to equip P_1_ with all the functionalities needed including high-level control for eventual clinical enteroscopy and descending coloscopy. This ambitious R&D program includes imaging sensing and smart LED lighting, human-body communication [20] to a wireless external console for live streaming.

Currently the P_1_ prototype has only a low-level open loop control. This requires major updating to high-level control essential for most of the essential functionalities including, forward/reverse active static locomotion which will require changing of the mounting angle of the legs by a dedicated mechanism, balloon cytology procurement (the mechanism of which has already been designed but still requires testing), together with development of an external wireless console for in-vivo operation of the fully developed platform.

We also need to adopt a fully compliant quality management system (QMS) for the development of SOFTIE to enable submission for CE marking, essential for clinical use. At this stage, we envisage testing the functionalized *final SOFTIE* prototype by in-vivo experiments in female in a dedicated already chosen in the veterinary unit of University of Edinburgh.

The intended users will be consultant clinical general surgeons (upper/lower) and clinical gastroenterologists. Both would operate the fully developed CE marked system from a console with many procedures being carried out by trained technicians or nurse practitioners under the remote guidance from a console by an expert clinician who will indicate when the need for biopsy of suspect lesion arises.

The clinical translation of SOFTIE would in the first instance replace WCE. In addition, it has the potential to replace flexible push endoscopy.

The clinical benefit from the SOFTIE platform primarily stems from obviating completely the need for flexible endoscopy procedures which cause discomfort, pain, and apprehension in the patients undergoing these procedures and which often necessitate sedation and analgesia.

In addition, SOFTIE will completely remove the aversion to invited national screening programs in the adult asymptomatic population for screening for the detection of early stage colorectal cancer, which is the reason accounting for the low compliance with the current mass screening of asymptomatic adult population.

When SOFTIE is fully developed, its clinical use will transform endoscopy suites as it will likely replace flexible endoscopes in the long term, with considerable cost savings to both national and private healthcare systems. Within any single endoscopy unit, specialist general surgeons/ gastroenterologists operating from a command console would oversee several endoscopy procedures carried out with SOFTIE, and indicate to the nurse practitioner/ technician the need for biopsy of suspect lesions. Perhaps more importantly, patients will lose their abhorrence and fear for all endoscopy procedures and hence subscribe far more readily to screening endoscopies for early detection of gastroesophageal and colorectal cancers.

### Supplementary Information

Below is the link to the electronic supplementary material.

Supplementary file1 (MP4 84747 kb)

## Appendix A: video contents

This video link provides the summary of this work including the experiments preformed on hybrid SOFTIE Prototype P_1_. https://youtu.be/u3LQWp4yI38
